# Identification of lenalidomide resistance pathways in myeloma and targeted resensitization using cereblon replacement, inhibition of STAT3 or targeting of IRF4

**DOI:** 10.1038/s41408-019-0173-0

**Published:** 2019-02-11

**Authors:** Yuan Xiao Zhu, Chang-Xin Shi, Laura A. Bruins, Xuewei Wang, Daniel L. Riggs, Brooke Porter, Jonathan M. Ahmann, Cecilia Bonolo de Campos, Esteban Braggio, P. Leif Bergsagel, A. Keith Stewart

**Affiliations:** 1 Division of Hematology, Mayo Clinic, Scottsdale, AZ USA; 2 Division of Biomedical Statistics and Informatics, Department of Health Sciences Research, Mayo Clinic, Rochester, MN USA; 3 Center for Individualized Medicine, Mayo Clinic, Rochester, MN USA

## Abstract

To understand immunomodulatory drug (IMiD) resistance in multiple myeloma (MM), we created isogenic human multiple myeloma cell lines (HMCLs) sensitive and resistant to lenalidomide, respectively. Four HMCLs were demonstrated to be resistant to all IMiDs including lenalidomide, pomalidomide, and CC-220, but not to Bortezomib. In three HMLCs (MM.1.SLenRes, KMS11LenRes and OPM2LenRes), *CRBN* abnormalities were found, including chromosomal deletion, point mutation, and low CRBN expression. The remaining HMCL, XG1LenRes, showed no changes in CRBN but exhibited CD147 upregulation and impaired IRF4 downregulation after lenalidomide treatment. Depletion of *CD147* in XG1LenRes and three additional HMCLs had no significant impact on MM viability and lenalidomide response. Further analysis of XG1LenRes demonstrated increased IL6 expression and constitutive STAT3 activation. Inhibition of STAT3 with a selective compound (PB-1-102) re-sensitized XG1LenRes to lenalidomide. Since XG1LenRes harbors a truncated IRF4 that is not downregulated by lenalidomide, we targeted IRF4/MYC axis with a selective inhibitor of the bromodomain of CBP/EP300 (SGC-CBP30), which restored lenalidomide response in XG1LenRes. This strategy also appeared to be more broadly applicable as SGC-CBP30 could re-sensitize two resistant HMCLs with low but detectable CRBN expression to lenalidomide, suggesting that targeting CBP/E300 is a promising approach to restore IMiD sensitivity in MM with detectable CRBN expression.

## Introduction

The immunomodulatory drugs (also known as IMiDs) thalidomide, lenalidomide, pomalidomide, and CC-220, play a pivotal role in the treatment of multiple myeloma (MM)^1,2^. While the majority of newly diagnosed MM patients respond to IMiDs therapy, most eventually develop resistance. The underlying mechanisms defining this non-responsiveness are still incompletely understood.

Cereblon (CRBN) was identified as the primary target of IMiDs^3^. CRBN was demonstrated to function as a substrate recognition component in a DCX (DDB1-CUL4-X-box) E3 protein ligase complex that mediates the ubiquitination and subsequent proteasomal degradation of target proteins. Binding of IMiDs alters the substrate specificity of CRBN, leading to the recruitment and degradation of proteins that regulate tumor proliferation, survival, and immune response^4^. In myeloma, upon IMiD treatment, IKZF1 and IKZF3 are recruited to CRBN- conjugated E3 protein ligase, become ubiquitinated, and degraded by the proteasome^5^. Downregulation of IKZF1/3 was demonstrated to induce downregulation of IRF4 and MYC^5–7^, two important proteins for myeloma proliferation and survival^8–10^. A recent study also demonstrated that CRBN promotes maturation of CD147–MCT1 proteins on MM cells and that IMiDs outcompete CRBN for binding to CD147 and MCT1, leading to destabilization of the CD147–MCT1 complex. The same study further showed that modulating CD147 and MCT1 expression by shRNA or overexpression affected MM cell viability and therefore proposed that destabilization of the CD147–MCT1 is associated with IMiD-mediated anti-myeloma activity^11^.

We, and others, previously demonstrated that low CRBN expression is associated with IMiD resistance^12–16^. Using a MM-targeting sequence panel, we recently found acquired mutations of CRBN and other genes in the CRBN E3 ligase complex or the downstream CRBN pathway in 22% of MM patients refractory to IMiDs^17^. This likely continues to underestimate CRBN pathway disruption in resistant disease since structural variation was not assessed. Notwithstanding, it is clear that some IMiDs resistant MM cases failed to demonstrate any abnormality in CRBN and its associated or downstream components, implying that CRBN-independent mechanisms of resistance exist. Indeed, in addition to CRBN deficiency or dysfunction, previous studies reported other mechanisms associated with IMiD resistance in MM cells, such as activation of Wnt signaling and the ERK pathway^18,19^. It therefore appears that multiple mechanisms are involved in IMiD resistance, but it is still unknown which mechanism is most prevalent and whether they are related.

In the present study, we established four lenalidomide-resistant human multiple myeloma cell lines (HMCLs) by culturing IMiD responsive HMCLs in the presence of lenalidomide for an extended time. Those resistant cell lines were studied along with their isogenic-sensitive lines to identify the genetic pathways underlying changes associated with resistance.

## Materials and methods

### Cells and reagents

All HMCLs used in this study were provided by Dr. Leif Bergsagel’s laboratory. All cell lines were fingerprinted using CNV analysis to confirm their identity as described^20^. Cells were maintained in RPMI-1640 media, supplemented with 5% fetal calf serum and antibiotics. All HMCLs tested *negative for mycoplasma* at the beginning and during the experiments (*using mycoplasma detection kit from Lonza*, Rockland, ME). XG1 was originally reported as an IL-6-dependent cell line^21^, however, the strain that we worked with also grew well in its absence. Antibodies against IRF4 (#4964), PARP (#9542), BIM (#2819), p-STAT3 (#9145), STAT3 (#9139), p-ERK1/2 (#9101), and ERK1/2 (#4695) were from Cell Signaling Technology (Danvers, MA). Anti-MYC (#ab32072) antibody was from Epitomics (Burlingame, CA). Anti-CRBN (#HPA045910) antibody was from Sigma-Aldrich (St. Louis, MO). Anti-IKZF3 (#img-6283a) was from Imgenex (Littleton, CO). Anti-IKZF1 (#sc-13039), Anti-CD147 (8D6) (#sc-21746), and MCT1 (H70) (#sc-50324) antibodies were from Santa Cruz Biotechnology (Dallas, TX). Lenalidomide (Len) and bortezomib (Bor) were from LC Laboratories (Woburn, MA). PB-1-102 (PB) was from Selleckchem (Houston, TX). CC-220 and SGC-CPB30 (SGC) was from MedChem Express (Monmouth junction, NJ). Human IL6 ELISA MAX^TM^ Deluxe set was from Biolegend (San Diego, CA).

### Establishment of lenalidomide-resistant HMCLs

Four lenalidomide-resistant HMCLs were generated in our laboratory by culturing lenalidomide-sensitive cell lines, MM.1S, KMS11, XG1, and OPM2, in the presence of lenalidomide for an extended period of time. Briefly, MM cells were cultured in the medium with a gradually increasing doses of lenalidomide (usually starting from 5 μM, up to 50 μM) until the stable resistant cells were generated. The identities of all resistant HMCLs generated were validated by fingerprint, showing identical fingerprints to their isogenic-sensitive cell lines. They also tested negative for mycoplasma.

### Knockout of *CD147 (BSG)* and CRBN using CRISPR-Cas9 technology

Lentiviral constructs expressing CRISPR-associated protein 9 (Cas9) and guide RNAs (gRNAs) originally generated from Feng Zhang’s lab^22^ were obtained from Addgene (Cambridge, MA). We first established the HMCLs stably expressing Cas9 by infection of HMCLs with lentivirus-expressing Cas9^23^. A total of four gRNAs targeting *CD147 and CRBN*, were selected from CRISPR pooled libraries generated from Dr. Fang Zhang’s and Dr. Jason Moffat’s labs, including: *CD147* #3 TTCACTACCGTAGAAGACCT; *CD147* #5 TGGAGCTGGTTGCCGTTGCAC; *CD147* #6 CGTCAGAACACATCAACGAG; *CRBN* #2 CCTTTGCTGTTCTTGCATAC. They were synthesized and cloned into the BbsI-digested plasmid containing the entire guide RNA scaffold. Lentivirus harboring non-targeting vector (Vec) and all gRNA expression constructs were generated and used to infect HMCLs. At day 3 after infection, puromycin was added to the media (5 μg/ml) in order to select infected cells. At days 7–14 after infection, cells infected with virus expressing *CD147* gRNAs were stained with anti-CD147 antibody, followed by sorting of CD147-negative cells (BD Biosciences FACS Aria III SORP Cell Sorter). The expression of CD147 in sorted cells was further evaluated by immunoblotting assay. For CRBN gRNA-transduced cells, the single clone that had no CRBN expression (validated by immunoblotting assay) was selected and expanded.

### Preparation of lentiviral virus expressing CRBN and infection of myeloma cells

The cDNA lentiviral constructs expressing human wild-type CRBN has been previously described^7^. The lentivirus harboring control vector and all expression constructs were generated to infect HMCLs. CRBN overexpression was confirmed by immunoblotting assay.

### Mate-pair sequencing

The high throughput genomic mate pair libraries from HMCLs were prepared by the Mayo Clinic Genome Core Facilities using the Illumina Mate Pair protocols and their available kit (Nextera Mate Pair Sample Preparation kit, Illumina)^10,24^, Then, two samples were run on one lane of an Illumina HiSeq2000 with 50 bp reads. The sequences were aligned to hg19 using BWA, and a BAM file containing only the clustered discordant reads with clustered mates was created. Breakpoints in the IRF4 locus with discordant reads were identified in the BAM file by visual inspection.

### Immunoblotting analysis

Western blot was performed according to the manufacturer’s protocol. Equal amounts of protein were subjected to sodium dodecyl sulfate polyacrylamide gel electrophoresis (SDS–PAGE) gels followed by transfer to PVDF membranes. Membranes were probed with primary antibodies overnight and then washed and incubated with horseradish peroxidase (HRP)-conjugated-secondary antibodies. Detection was performed by the enhanced chemical luminescence (ECL) method.

### IL-6 ELISA assay

IL6 in the cell cure media was measured by ELISA assay (Human IL-6 ELISA kit was from Biolegend). Briefly, MM cells were cultured with or without lenalidomide treatment for 48 h. The cell culture media was harvested, diluted, and measured according to the manufacture’s instruction.

### Cell viability assay

Cell viability or cell growth was measured by 3-(4,5-dimethylthiazol)-2,5-diphenyl tetrazolium (MTT) dye absorbance according to the manufacturer’s instructions (Boehringer Mannheim). Each experimental condition was performed in triplicate and was repeated at least twice.

### Array-based Comparative Genomic Hybridization (aCGH)

DNA was extracted from mononuclear cells using Puregene kit (Qiagen) and the manufacturer’s recommended protocol. All HMCLs were run using the Human 400K microarray (Agilent Technologies). The digestion, labeling, and hybridization steps were performed as previously described with minor modifications^25^. Copy number abnormalities (CNA) were calculated using two-probe and 0.2 log2 filters and aberration detection module (ADM)-1 algorithm^26^ with a threshold of 9.0.

### Targeted DNA sequencing

Targeted sequencing was performed on the Ion Torrent semiconductor sequencing platform (PGM, Life Technologies, Carlsbad, CA) and analyzed as described previously^23,27^. Overall, we obtained an average coverage depth of ×500. Variants with a mapping quality <20 or read depth <10X were removed. Variants of significant interest were visually inspected using the Integrative Genomics Viewer^28^.

### mRNA sequencing (mRNA-seq)

MM cells were harvested and total RNA was prepared using RNeasy plus kit under the protocols of the manufacturer (Qiagen, Valencia). mRNA-seq experiment was performed and analyzed as described previously^23^ at the medical genome facility in Mayo Clinic, Rochester. RNAseq data was processed using Mayo RNA-Seq Bioinformatics workflow MAP-RSeq^29^, including read alignment against human hg19 genome build (by TopHat v2.0.12) and read count quantification for genes (by featureCount v1.4.4). Differential expression analysis was then performed with edgeR v2.6.2^30^ to identify genes with altered expression between XG1 and XG1res. Genes were determined as differentially expressed based on the criteria (FDR < = 0.05). Up-regulated and down-regulated genes were then separately submitted to Ingenuity Pathway Analysis (http://www.ingenuity.com/) for identifying enriched pathways.

## Results

### Resistance to IMiDs in MM cell lines is usually associated with CRBN abnormalities

**Fig. 1:**
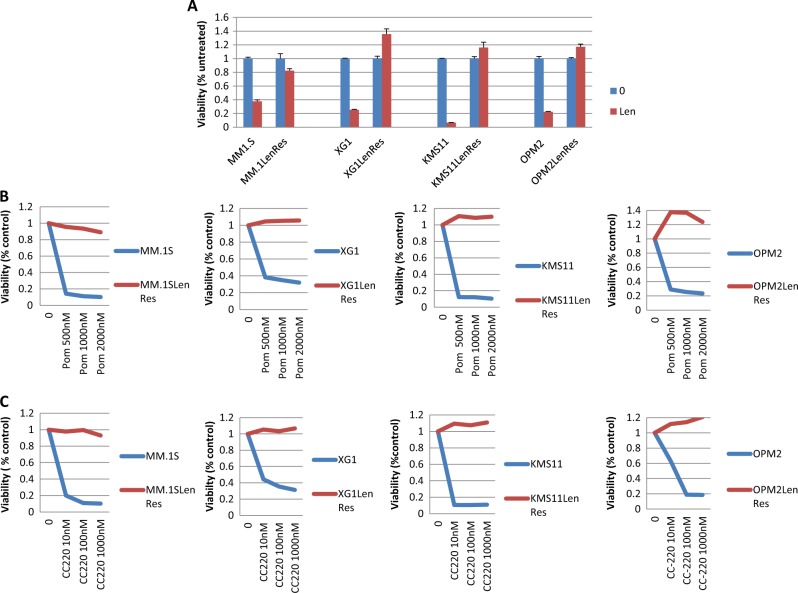
IMiD-resistant HMCLs were generated after prolonged lenalidomide exposure. Four IMiD-resistant HMCLs were generated from parental MM1.S, KMS11, OPM2, and XG1 after prolonged lenalidomide exposure and each was demonstrated to be resistant to all IMiDs including (**a**) lenalidomide (Len), (**b**) Pomalidomide (Pom) and (**c**) CC-220 in a representative MTT assay (day 5 after treatment)

Four HMCLs with acquired resistance to lenalidomide were established in this study by culturing their isogenic lenalidomide-responsive HMCLs (MM.1S, KMS11, XG1, and OPM2), in the presence of lenalidomide for an extended period of time. After fingerprinting confirmation of cell line origin, responses of the derived cell lines to more potent IMiDs (pomalidomide and CC-220) and proteasome inhibition (bortezomib, as a control) were tested. All four isogenic resistant lines were resistant to each of the IMiDs (Fig. 1a–c), but not to Bortezomib (supplementary Figure 1).

**Fig. 2:**
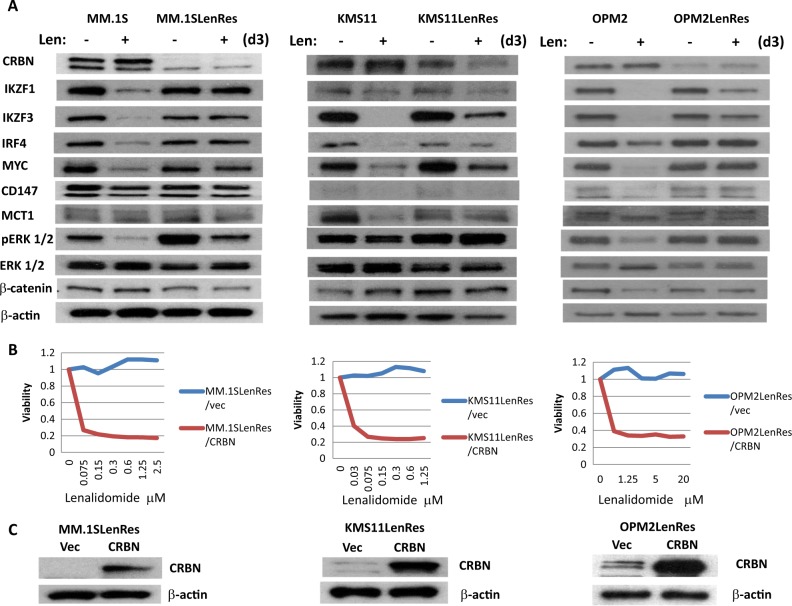
Acquired resistance to IMiDs in three of four resistant HMCLs is associated with reduced CRBN expression and dysfunction. **a** Compared to their isogenic-sensitive lines, three resistant HMCLs (MM.1SLenRes, KMS11LenRes, and OPM2LenRes) show reduced CRBN expression and impaired IKZF1/3, IRF4, and MYC downregulation after lenalidomide treatment on immunoblotting. **b** and **c** Introduction of exogenous CRBN into those resistant HMCLs restored their sensitivity to lenalidomide. Briefly, each resistant line was infected by lentivirus harboring control vector (Vec) or *CRBN-*expressing cassette, followed by MTT assay of lenalidomide response (**b**) and immunoblotting assays of CRBN expression (**c**)

To identify the underlying mechanisms for resistance, immunoblotting assay was initially performed to evaluate the proteins that have been previously reported to be associated with either IMiD response or resistance, including CRBN, IKZF1/3, IRF4, MYC, CD147, MCT1, CTNNB1, and ERK1/2. Three of the four resistant HMCLs (MM.1SLenRes, KMS11LenRes, and OPM2LenRes) were identified with a significant decrease or absence of CRBN protein expression when compared to their isogenic IMiD-sensitive lines (Fig. 2a). Accordingly, CRBN-mediated degradation of IKZF1 and IKZF3 and down-regulation of IRF4 and MYC upon lenalidomide treatment in those cells was abrogated or substantially reduced. Consistent with a previous study, an increased activation of ERK was detected in MM.1SLenRes^19^. When wild type-CRBN was re-introduced into the three resistant cell lines, the sensitivity to lenalidomide was completely restored (Fig. 2b, c). Two of those resistant cells were further analyzed by aCGH and targeted DNA sequencing, demonstrating that MM.1SLenRes carries both a deletion and a mutation in the remaining copy of CRBN, while KMS11LenRes has a CRBN deletion (Table 1, supplementary Figures 2–3). No other mutations were detected in genes that encode CRBN-binding proteins or downstream components.

**Table 1:** Genomic analysis of IMiDs-resistant HMCL

HMCLs	aCGH	Targeted DNA sequencing
MM.1S parental	1 copy CRBN on a 2N karyotype	Negative
MM.1SLenRes	1 copy CRBN on a 2N karyotype	CRBN Mutation
		SNV: Ser 271 Phe (24%)
		Truncation: Trp399 (8%)
		Splice mutation (24%)
KMS11 parental	1 copy CRBN on a 2N karyotype (subclonal), 3 copy on a 4N karyotype	Negative
KMS11LenRes	0 copy CRBN on a 2N karyotype (subclonal), 1 copy on a 4N karyotype	Negative

### XG1LenRes has altered CD147 expression but depletion of *CD147* had no significant impact on MM viability and lenalidomide response

**Fig. 3:**
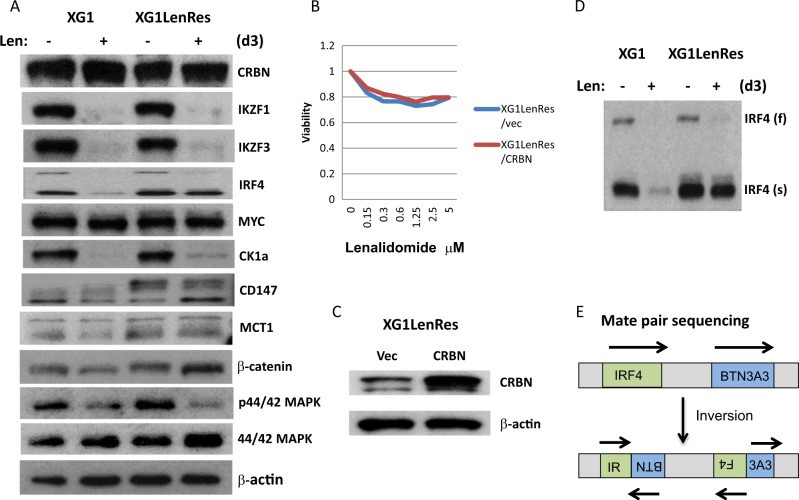
XG1LenRes demonstrated altered CD147 expression and impaired lenalidomide-mediated downregulation of IRF4 (truncated form). **a** Immunoblotting of XG1 and XG1LenRes demonstrated that XG1LenRes has normal CRBN expression and has an altered CD147 expression compared with XG1. **b** Introduction of exogenous CRBN into XG1LenRes did not restore its sensitivity. **c** In addition to a full-length (f) IRF4, a short (s) IRF4 protein was detected from both XG1 and XG1LenRes. Upon lenalidomide treatment, shorted IRF4 in XG1LenRes was not downregulated as well as in XG1. **d** Mate pair sequencing data suggested that the *IRF4* gene in both XG1 and XG1LenRes has an inversion mutation, which is likely to produce a short C-terminal-truncated IRF4 protein

Compared with the other three resistant HMCLs, XG1LenRes still has abundant CRBN expression after acquiring lenalidomide resistance (Fig. 3a). Consistently, lenalidomide-induced IKZF1/IKZF3 degradation in XG1LenRes is identical to its isogenic line, XG1, and introduction of exogenous CRBN failed to restore lenalidomide sensitivity (Fig. 3b, c), suggesting that lenalidomide resistance is mediated downstream of CRBN or through CRBN-independent mechanism. In addition to a full-length of IRF4, a short IRF4 protein was also detected from both sensitive and resistant XG1 cells by immunoblotting (Fig. 3d). By analysis of mate pair whole genome sequencing of XG1, we identified that one allele of IRF4 gene is inverted, which is likely producing a short, c-terminal-truncated IRF4 protein (Fig. 3e and supplementary Figure 4). Accordingly, the short IRF4 protein was not detected by an antibody targeting the C-terminal region of IRF4 (supplementary Figure 5). Upon lenalidomide treatment, both full length and truncated IRF4 were substantially downregulated in XG1 cells whereas only full- length of IRF4 was substantially downregulated in XG1LenRes (Fig. 3d). To understand whether this truncated IRF4 contributed to IMiDs resistance, we cloned the cDNA of this truncated IRF4 and expressed it in an IMiD-sensitive cell line (KMS11) that only has a full length of IRF4 expression. It demonstrated that truncated IRF4 significantly reduced the sensitivity of KMS11 to lenalidomide, but it did not completely block lenalidomide activity (supplementary Figure 6).

**Fig. 4:**
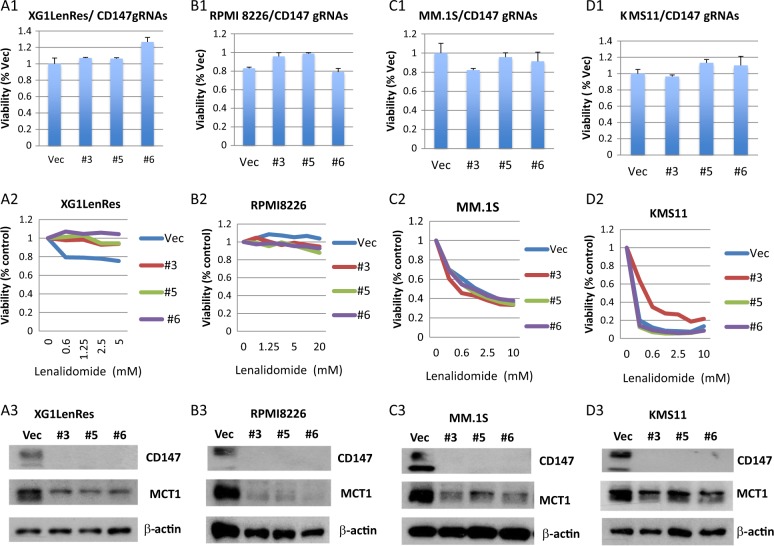
Depletion of *CD147* did not significantly affect myeloma cell line viability and sensitivity to lenalidomide. **a**–**d** Cell viability, lenalidomide response, and expression of CD147/MCT1 (from up to down) were measured in XG1LenRes (**a**) and three additional HMCLs (**b**–**d**) to compare control cells (vector) to the cells after depletion of CD147 by CRSPR cas9 technology with three gRNAs (#1–#3). Cell viability and drug response were set up either in the absence or presence of lenalidomide at indicated concentration, measured at day 5 after treatment. The results from a representative experiment with triplicates are shown

In addition to the short form of IRF4, CD147 was also upregulated in XG1LenRes. We investigated if increased CD147 expression contributes to the acquired resistance in XG1LenRes. CRISPR-cas9 technology was used to knockout the endogenous *CD147* in XG1LenRes. As shown in Fig. 4a, depletion of *CD147* with three different gRNAs did not reduce cell proliferation nor did it restore lenalidomide sensitivity. Consistent with a previous study which demonstrated that CD147 regulates MCT1 expression^31^, *CD147* depletion induced a substantial reduction of MCT1 expression in XG1LenRes. We further expanded our work to three additional HMLCs including two IMiDs sensitive cell lines (KMS11 and MM.1 S) and one resistant cell line (RPMI8226). Depletion of *CD147* in those three HMCLs also failed to significantly affect MM cell viability and sensitivity to lenalidomide (Fig. 4b–d).

### IL6 upregulation and STAT3 activation is identified as a resistance mechanism to IMiDs

**Fig. 5:**
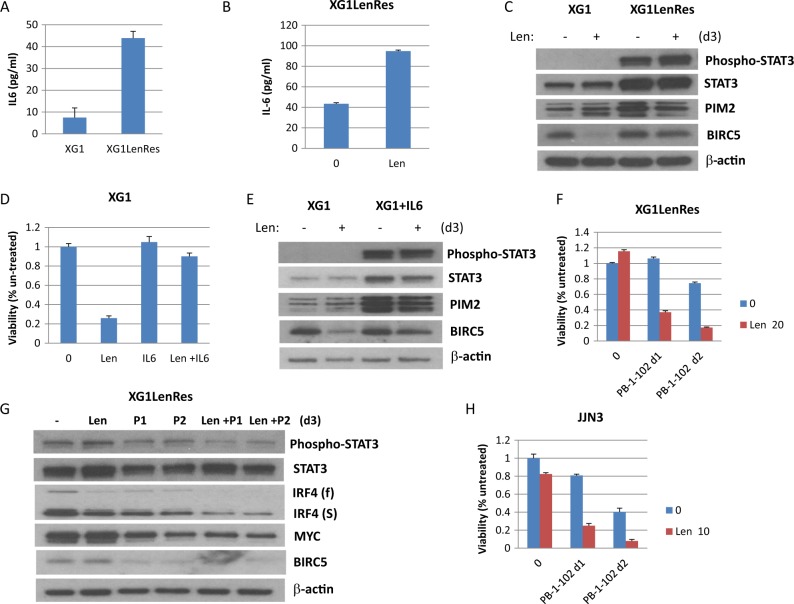
IL6 autocrine and STAT3 activation was prominent in XG1LenRes and inhibition of STAT3 sensitized XG1LenRes to lenalidomide. **a** An increased IL6 secretion was detected from the cell culture medium of XG1LenRes. IL6 secretion in this resistant cell line was enhanced by lenalidomide treatment (**b**). **c** Immunoblotting of XG1 and XG1LenRes demonstrated STAT3 activation and increased downstream STAT3 target gene expression in XG1LenRes. **d** and **e** Adding exogenous IL6 (1 ng/ml) to XG1 culture resulted in lenalidomide resistance (by MTT assay at day 5 after treatment) and STAT3 activation (Immunoblotting assay at day 3 after treatment). **f** and **g** Co-treatment of XG1LenRes with lenalidomide (Len 20 μM) and a selective STAT3 inhibitor (PB-1-102 or P) at increasing doses (d1: 5.5 μM; d2: 6.0 μM) generated a synergic anti-myeloma activity (at day 5 after treatment) and downregulation of IRF4 and MYC (at day 3 after treatment). **h** Inhibition of STAT3 with an increasing doses of PB-1-102 (d1: 7 μM; d2: 8 μM) also overcame the intrinsic resistance to lenalidomide in JJN3 cells, which was identified to harbor-activated STAT3

To further explore the underlying mechanisms mediating IMiD resistance in XG1LenRes, mRNAseq was performed in sensitive and resistant cells. Compared with parental XG1, the expression of 4764 genes was substantially altered (at least 1.5-fold change) in resistant cells including 1297 upregulated and 3467 downregulated genes (supplementary table 1). Pathway analysis indicated that JAK/ STAT signaling was one of the top canonical pathways enriched with up-regulated genes (supplementary table 2). Among the most up-regulated genes we identified interleukin 6 (*IL6*) and *STAT3*. To assess whether autocrine production of IL6 and activated STAT3 is associated with IMiD resistance, we performed ELISA and immunoblotting assays to measure IL6 secretion and STAT3 activation in XG1LenRes cells. As shown in Fig. 5a, an increase in IL6 was detected in the culture media of XG1LenRes compared to XG1. Surprisingly, lenalidomide treatment further enhanced IL6 production in XG1LenRes (Fig. 5b). Consistent with mRNAseq data, *STAT3* and its downstream genes (*PIM2* and *BIRC5*) expression were increased in XG1LenRes). A highly activated STAT3 was detected in XG1LenRes but not in XG1 (Fig. 5c). Similarly, when IL6 was supplemented in the culture media of isogenic parental sensitive XG1, XG1 became resistant to lenalidomide (Fig. 5d) with an activated STAT3 and increased downstream gene expression (Fig. 5e). To further determine if activation of STAT3 is involved in resistance to IMiDs in this cell line, a selective STAT3 inhibitor (PB-1-102) was then employed which re-sensitized XG1LenRes to lenalidomide and induced a synergistic anti-myeloma activity and downregulation of IRF4 (both full length and short form) and MYC expression (Fig. 5f, g).

We explored the role of STAT3/IL-6 in a broader array of MM cell lines. Three of seven HMCLs with primary IMiD resistance were demonstrated to have activated STAT3 (supplementary Figure 7). Synergistic anti-myeloma activity was also achieved with lenalidomide and PB-1-102 co-treatment in one representative tested cell line (Fig. 5h). To determine clinical relevance as to whether upregulation of IL6 or STAT3 was clinically significant, we used the MMRF coMMpass data and noted that MM samples with IL6 expression and, or, high STAT3 expression were associated with a shorter response (supplementary Figure 8).

### Targeting IRF4 by inhibition of the bromodomain (BRD) of CBP/EP300 re-sensitizes IMiD-resistant HMCLs to lenalidomide

**Fig. 6:**
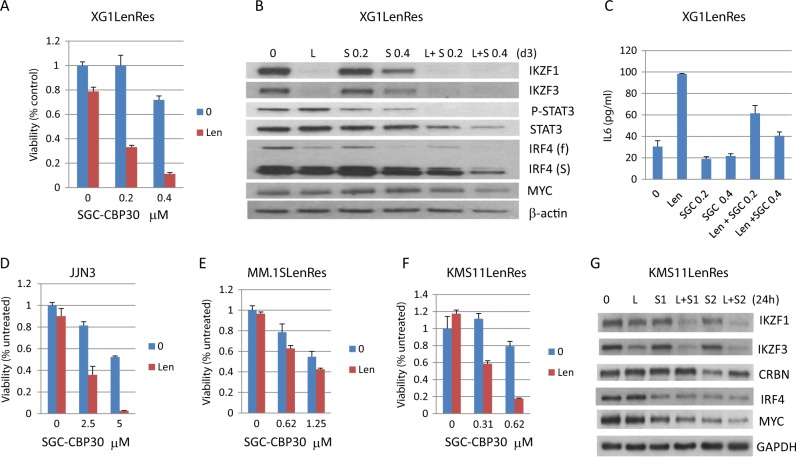
Targeting IRF4 by inhibition of the bromodomain of CBP/EP300 re-sensitized XG1LenRes and several other resistant HMCLs to lenalidomide. **a** The IMiD-resistant cell line, XG1LenRes, was analyzed by MTT assay after co-treatment with lenalidomide and the indicated doses of SGC-CBP30. The response to lenalidomide was substantially restored after SGC-CBP30 was added. **b** Immunoblotting assays detected the changes of IRF4, MYC, and STAT3 after treatment with either lenalidomide and SGC-CBP30 (S, 0.2 and 0.4 μM) alone or combination (day 3). **c** SGC-CBP30 treatment also reduced IL6 levels in the culture media of XG1LenRes at day 2 after treatment (by ELISA assay). **d**–**f** The IMiD-resistant HMCLs (JJN3, MM.1SLenRes, and KMS11LenRes) were analyzed by MTT assay at day 5 after co-treatment with lenalidomide (10 μm) and the indicated doses of SGC-CBP30. **g** Immunoblotting analysis was performed at 24 h after co-treatment of KMS11LenRes with Lenalidomide (10 μm) and SGC-CBP30 (S1: 0.5 μM and S2: 0.625 μM)

Since IRF4, especially the truncated IRF4, was not substantially downregulated in XG1LenRes compared with sensitive XG1 after lenalidomide treatment, we decided to look at whether targeting IRF4 could also re-sensitize XG1LenRes to lenalidomide. The small molecule (SGC-CBP30) has a selective affinity for the BRD of CBP/EP300^32^ and has recently been demonstrated to target the IRF4 network in MM^33^. As shown in Fig. 6a, the combination of SGC-CBP30 and lenalidomide generated a substantial synergy in reducing myeloma cell viability. Consistent with a previous report^33^, SGC-CBP30 downregulated IRF4 in a dose-dependent manner (Fig. 6b). XG1LenRes co-treatment with lenalidomide and SGC-CBP30 induced the most effective downregulation of IRF4 (especially truncated IRF4) and MYC (Fig. 6b). In order to know whether SGC-CBP30 treatment has an effect on IL6 autocrine secretion in XG1LenRes cells, we measured IL6 in the culture media of XG1LenRes in the absence and presence of drug treatment. As shown in Fig. 6c, SGC-CBP30 treatment also reduced IL6 autocrine production from XG1LenRes in either the absence or presence of lenalidomide. Accordingly, STAT3 activation was inhibited by SGC-CBP30 treatment (Fig. 6b).

In order to generalize applicability of SGC-CBP30 we treated another six IMiD-resistant HMCLs, including the three CRBN-deficient resistant lines generated in this study (MM.1SLenRes, KMS11LenRes, and OPM2LenRes), two intrinsically resistant HMCLs (JJN3 and OCI-MY5) and one resistant cell line we had generated in house by *CRBN* knockout (MM1.R CRBN^−^). In the HMCL that expresses the most abundant CRBN (JJN3), co-treatment with lenalidomide and SGC-CBP30 induced a significantly synergistic anti-myeloma activity (Fig. 6d). In contrast, in the HMCLs that have an absence of CRBN expression and have CRBN mutation (MM.1R CRBN^−^ and MM.1SLenRes) no significant synergies were detected (supplementary Figure 9A and Fig. 6e). In three resistant HMCLs with low but measureable CRBN expression (KMS11LenRes, OPM2LenRes, and OCIMY5), KMS11LenRes and OPM2LenRes regained responsiveness to lenalidomide after co-treatment with SGC-CPB30 (Fig. 6f and supplementary Figure 9B–C). The lenalidomide resensitization is not related to an increase of CRBN expression in KMS11LenRes (Fig. 6g), but appears to be associated with IKZF1/IKZF3, IRF4, and MYC downregulation. Finally, we demonstrated that SGC-CBP30 also increased the sensitivity to lenalidomide in three IMiD-sensitive HMCLs (supplementary Figure 9D–F).

## Discussion

In order to better elucidate the underlying mechanisms by which myeloma cells develop IMiD resistance, we established four resistant HMCLs by culturing their isogenic sensitive cell lines in the presence of lenalidomide for an extended period of time. Consistent with our recent clinical data that showed CRBN mutations in relapsed patient samples^17^, we demonstrated that 3 of 4 resistant HMCLs have reduced or absent CRBN expression after acquiring resistance to IMiDs and two tested cell lines have either *CRBN* deletion or mutations. Although we also detected activation of ERK and WNT signaling in some resistant cell lines that have reduced CRBN expression, introduction of exogenous wild-type CRBN into those cell lines restored their sensitivity to lenalidomide, suggesting that CRBN deficiency and dysfunction remains the major source of resistance in MM.

The outlier was the cell line XG1LenRes which harbored no CRBN deficiency or dysfunction, suggesting that resistance in this cell line is mediated by a CRBN-independent mechanism. Although we found that lenalidomide-induced CD147downregulation in IMiD-sensitive cell lines, depletion of *CD147* in XG1LenRes and three additional HMCLs did not change MM viability and IMiD response. Moreover, *CD147* depletion was demonstrated to substantially decrease MCT1 expression in those MM cells, suggesting that lenalidomide-mediated anti-myeloma activity detected from the in vitro model is not associated with disruption of CRBN-CD147-MCT1 axis.

**Fig. 7:**
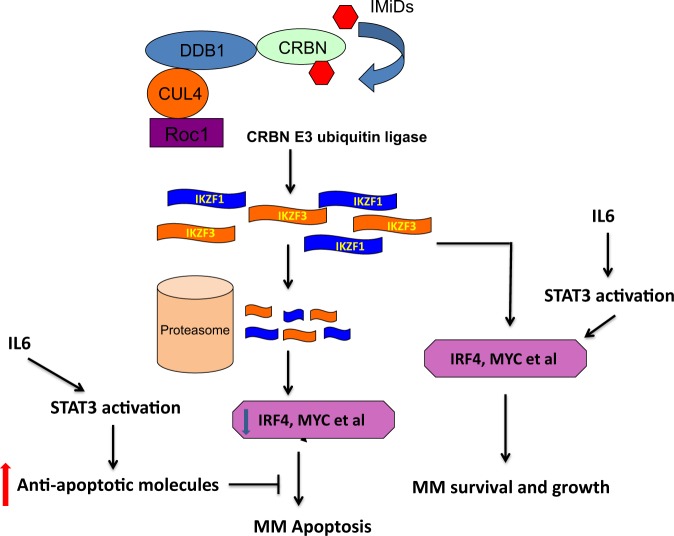
Proposed mechanism of action of autocrine IL6 and activation of STAT3 on IMiDS mediated anti-myeloma activity. IKZF1/IKZF3 regulate IRF4, MYC, and other genes to support MM cell proliferation and survival. Upon IMiDs treatment, IKZF1/IKZF3 are recognized by CRBN E3 ubiquitin ligase, degraded in the proteosome, resulting in downregulation of IRF4, MYC, and other genes that further induce inhibition of MM cell growth and apoptosis. Autocrine IL6 activates STAT3 pathway to upregulate anti-apoptotic molecules. Therefore, STAT3 activation may antagonize the apoptotic signals induced by IMiD-mediated IKZF1/IKZF3 and IRF4 downregulation. STAT3 may also regulate IRF4, MYC, and other gene expression in tumor cells to support MM growth and survival

By performing mRNAseq analysis, we identified upregulation of both IL6 and STAT3 in XG1LenRes when compared with parental cells, correlating with a highly activated STAT3/JAK signaling. We further demonstrated that XG1LenRes has IL6 autocrine production, which was surprisingly enhanced by lenalidomide treatment. Inhibition of STAT3 sensitized XG1LenRes to lenalidomide whereas adding IL6 to the wild type XG1 induced lenalidomide resistance, indicating that IL6 autocrine and STAT3 activation are the source of lenalidomide resistance in XG1LenRes. We went on to show that some HMCLs with intrinsic resistance to IMiDs also have activated STAT3, including JJN3. STAT3 inhibition in JJN3 enhanced its response to lenalidomide. Downstream genes activated by STAT3 have been associated with tumor proliferation and survival, including PIM1/PIM2, MYC, BIRC5 and BCL2L1^34–39^. Accordingly, mRNAseq data or immunoassay indicated that BIRC5, PIM2, and BCL2L1 were elevated in XG1LenRes (when compared with XG1). We assume that those anti-apoptotic molecules induced by STAT3 activation may antagonize the apoptotic signals induced by IMiD-mediated IKZF1/IKZF3 and IRF4 downregulation (Fig. 7). A recently published study demonstrated that activated STAT3 is involved in IRF4 regulation to support the survival of anaplastic large cell lymphomas^40^, implying that STAT3 may also regulate IRF4 expression in other tumor cells. In XG1LenRes, IRF4, especially truncated IRF4, was not downregulated in XG1 cells after lenalidomide treatment, suggesting that resistance to IMIDs in XG1LenRes could also be a result from STAT3-mediated IRF4 dysregulation (Fig. 7).

Using a small compound (SGC-CBP30) that selectively targets the IRF4 network in MM cells, we demonstrated that the resistance to lenalidomide could be overcome after co-treatment of XG1LenRes with SGC-CBP30 and lenalidomide. SGC-CBP30 is an inhibitor of the BRD-containing transcription factors CREBBP (CBP) and EP300. CBP and EP300 function as transcriptional co-activators via acetylation of histones and transcription factors^41^. Inhibition of CBP/EP300 has been demonstrated recently to target the IRF4 super enhancer and MYC regulatory region in MM cells^33^. Consistent with this result, we found that IRF4 (both full-length and short form) and MYC were downregulated after inhibition of CBP/EP300 by SGC-CBP30 in XG1LenRes. Interestingly, we found that SGC-CBP30 also inhibits IL6 autocrine production and STAT3 activation in XG1LenRes, suggesting that the SGC-CBP30-induced recovery of IMiD sensitivity might be mediated not only by direct inhibition of CBP/EP300 in IRF4/MYC regulatory region, but also by indirectly inhibiting IL6 expression and STAT3 activation.

By further expanding the SGC-CBP30 study to other HMCLs, we demonstrated that SGC-CBP30 also increased IMiDs sensitivity in three sensitive cell lines and re-sensitized several IMiD-resistant HMCLs to lenalidomide. Although we demonstrated that CRBN is required for SGC-CBP30-mediated resensitization, for some resistant HMCLs with a low but detectable CRBN expression, SGC-CBP30-exposed cells regained response to lenalidomide through a non-CRBN-related mechanism. One possible explanation is that inhibition of CBP/EP300 results in IRF4/MYC downregulation which synergizes with IMiDs action in the same cells. Another possibility is that SGC-CBP30 treatment would change the chromatin structure in the IRF4 and MYC regulatory regions or regulate other genes in IMiDs-mediated activity, therefore inducing increased sensitivity to IMiD-induced signals (such as downregulation of IKZF1/IKZF3).

In summary, we demonstrated that CRBN deficiency and mutation remains the most common mechanism associated with acquired resistance to IMiDs in MM. We also identified a novel mechanism of resistance associated with IL6 and activation of STAT3. Our study further demonstrated inhibition of CBP/EP300 and IRF4 as a promising strategy to overcome IMiD resistance.

## Supplementary information

Supplementary Figures 1-9

mRNA-seq analysis identified the genes deferentially expressed in XG1 and XG1LenRe

Top canonical pathways enriched with the genes up-regulated in XG1LenRes

Author Meta Data
