# Supplementary material for: Identification of lenalidomide resistance pathways in myeloma and targeted resensitization using cereblon replacement, inhibition of STAT3 or targeting of IRF4
Source: Blood Cancer J. 2019 Feb 11;9(2):19. doi: 10.1038/s41408-019-0173-0 (PMC6370766; doi:10.1038/s41408-019-0173-0)
Supplement: Supplementary file 1 — Supplementary Figures 1-9 [file 41408_2019_173_MOESM1_ESM.docx]

Supplementary figure 1. Four IMiD resistant HMCLs were generated from MM.1S, KMS11, OPM2 and XG1 after a prolonged lenalidomide exposure and all of them were demonstrated to respond to bortezomib by MTT assay at day 3 after treatment.

Supplementary figure 2. aCGH analysis of CRBN copy numbers in MM.1S and MM.1SLenRes. The results were compared with the MM.1Sres from Dr. Orlowski’s lab (Blood.2011 Nov3: 4771-9).

Supplementary fagure 3. aCGH analysis of CRBN copy numbers in KMS11 and KMS11LenRes cells. Compared with original KMS11, CRBN copy number changes were identified in parental KMS11 cells (used to generate KMS11LenRes) and KMS11LenRes.

Supplemental figure 4. Mate pair sequencing data of XG1 was analyzed and the orientation of the mate pair reads is consistent with a chromosomal inversion on chromosome 6 between IRF4 and BTN3A3.

Supplementary figure 5. Immunoblotting assay was performed on XG1 after treatment without and with lenalidomide treatment. A full length and a short IRF4 were detected by anti-IRF4 antibody against N-terminal of IRF4 , only full length of IRF4 was detected by anti-IRF4 antibody that target C-terminal of IRF4.

Supplementary figure 6. The sequence of mRNA encoding truncated IRF4 fusion protein was identified by analysis of mRNAseq data of XG1 and XG1res (A). cDNAs for this truncated IRF4 were prepared by reverse transcription of total RNA from XG1 and XG1res cells, followed by PCR amplification using the primers ( Forward primer 5’AATAATTCTAGAATGAACCTGGAGGGCGGCGGC3’ and Reverse primer 5’ACTGAAGGATCCTCAGAAATTCCCTCCTCGGCCATTTTCAC3’). cDNA was cloned into lentiviral expression vector and it was verified by DNA sequencing. The lentivirus harboring empty vector or truncated IRF4 from XG1 (S1) and XG1LenRes (S2) were prepared and used to infect KMS11 cells. The expression of truncated IRF4 was detected by western blot (B) and lenalidomide response of those infected cells was measured by MTT assay.

Supplementary figure 7. STAT3 status was analyzed by immunoblotting assay in eight IMiDs resistant MM cell lines and three of them were shown to have activation of STAT3 (phosphorylated STA3).

Supplementary figure 8. These plots were prepared using MMRF CoMMpass data (Explore 1A13) and analysis software. They show the Kaplan-Meier curves for selected clinical endpoints with censoring. The plots show the estimated probability over time for all patients in the dataset. Selecting a single cohort will show the differential probabilities between the patients belonging to the selected cohort and patients excluded from the selected cohort. A test of equal hazards between groups is performed and the p-value for the log-rank is displayed along with the hazard ratio between groups.

Supplementary figure 9. The response to lenalidomide was measured in three resistant (A-C) and three sensitive (D-E) cell lines without or with cotreatment with SGC-CBP30 and lenalidomide. OPM2LenRes regained partial response to lenalidomide after cotreatment. The resistant cell lines with CRBN depletion (MM1.R CRBN k/o) or with very low CRBN expression (OCIMY5) did not show a significant resensitization after cotreatment.
